# National Practice in Antibiotic Prophylaxis in Breast Cancer Surgery

**DOI:** 10.4021/jocmr1642w

**Published:** 2013-12-13

**Authors:** Aydan Eroglu, Durdu Karasoy, Halil Kurt, Semih Baskan

**Affiliations:** a Department of General Surgery and Surgical Oncology, Ankara University Medical School, Ankara, Turkey; b Department of Statistics, Hacettepe University Faculty of Science, Ankara, Turkey; c Department of Infectious Diseases and Clinical Microbiology, Ankara University Medical School, Ankara, Turkey; d Department of General Surgery, Ankara University Medical School, Ankara, Turkey

**Keywords:** Breast cancer, Surgery, Antibiotic, Prophylaxis, National survey

## Abstract

**Background:**

Although breast cancer surgery is regarded as a “clean” surgery, surgical site infection (SSI) rates are higher than expected. There is no consensus regarding the use of antibiotic prophylaxis in elective breast surgery. The nationwide survey was conducted to determine the trend of antibiotic prophylaxis in breast cancer among Turkish surgeons.

**Methods:**

The survey was sent to surgeons who are member of Turkish Surgical Association (TSA) via e-mail from TSA web address. A 15 item web-based survey consisted of surgeon demographics and the use of prophylactic antibiotic in patients with risk factors related to SSI.

**Results:**

The number of completed questionnaires was 245. The most common antibiotic used was first generation of cephalosporins. A majority of respondents indicated that prophylaxis was preferred in patients with high risk of SSI including preoperative chemotherapy or radiotherapy, older age, diabetes mellitus, immunodeficiency, immediate reconstruction (P < 0.05). However, the use of drain did not significantly influence antibiotic prophylaxis (P = 0.091).

**Conclusions:**

The use of prophylactic antibiotic was strongly dependent on the presence of some risk factors; however, the variation in current practice regarding antibiotic prophylaxis demonstrated a lack of its effect on preventing SSI after breast cancer surgery.

## Introduction

It is well known that the reduction of post-operative infection rate is established in clean-contaminated wounds by using the antibiotic prophylaxis. The rate of wound infection after clean surgery is approximately 1.5%. The rates of surgical site infection (SSI) after breast surgery are higher than other clean wounds in which the rate of infection is less than 5% [1-4].

There has been no consensus regarding the use of antibiotic prophylaxis in breast cancer surgery because of conflicting results. Despite lack of evidence of efficacy of perioperative antibiotic prophylaxis, some surgeons have used the antibiotic for breast cancer surgery and they have reported that antibiotic prophylaxis reduces the postoperative SSI rate [1, 2, 4-7]. However, other studies have showed that there is no significant reduction in SSI rate with the use of antibiotic after breast surgery [3, 8-11].

Recent Cochrane studies [12] and meta-analysis by Tejirian et al [4] revealed that prophylactic antibiotic reduce the risk of SSI in patients undergoing breast cancer surgery. Their meta-analysis showed that prophylactic antibiotics reduce the risk of postoperative wound infections after breast surgery. The authors also indicated that decreasing SSIs could be critical not only for cosmesis, also to prevent delays in adjuvant therapy or in any additional surgical definitive procedure. However, in a new meta-analysis, antibiotic prophylaxis in breast surgery was not found to be an independent protective factor for SSI [13]. It is clear that the studies remain conflicting results rather than conclusive. On the other hand, today, there are a few published data concerning the use of antibiotic prophylaxis in breast surgery as the nationwide survey [14, 15]. Therefore, we have conducted a nationwide survey regarding antibiotic prophylaxis. The main objective of the survey is to establish the current practice of the use of perioperative antibiotic in elective breast surgery as well as surgeons’ characteristics.

## Methods

The survey content and distribution were approved by Turkish Surgical Association (TSA)’s committee and director. The members of the society were contacted via e-mail addresses.

Approximately 2,700 general surgeons are member of TSA, but it is not known that how many surgeons are interested in breast surgery. Therefore the survey was sent to all general surgeons who are member of TSA. It was distributed via an e-mail containing a hyperlink with a short letter from TSA website (www.turkcer.org). The intent of the request letter was to complete the questionnaire if the surgeon is interested in breast cancer surgery. A total of 2,700 questionnaires were sent out. A reminder e-mail was sent after 3 weeks. In addition, we attempted to increase response rates by placing phone calls to non-respondents.

The survey consisted of 15 multiple-choice questions about surgeon demographics including practice volume, practice setting, prophylactic antibiotic use with and without immediate reconstruction, use of surgical drains, SSI rate despite of antibiotic, type of microorganism as the most cause of SSI, type of antibiotic, duration of the antibiotic. The study data were collected by Pleksus Informatics Technologies (www.pleksus.com) on behalf of TSA. The reported responses were evaluated as percentage and then subject to the statistical analysis.

The statistic was performed according to the proportions of respondents. The results were analyzed using chi-square test with significance determined at P < 0.05. The statistical analysis was performed by SPSS software (Chicago, IL).

## Results

A total of 2,700 surveys were delivered electronically via website of TSA. A total of 245 completed questionnaires were returned from the general surgeons interested in breast surgery among the members of TSA.

A majority of respondents (52.7%) described in their practice as university or academic affiliated. Others indicated being in state hospital or private practice (16.6% and 30.7%, respectively). As seen in Table 1, a majority of respondents (64.1%) reported their years in practice as ≥ 10 years, while this duration was < 10 years in others (34.9%).

**Table 1:** Characteristics of Surgeons

Characteristic	%*
Practice description	
University affiliated	30.4%
State education and research	22.3%
State hospital	16.6%
Private	30.7%
Years in practice as surgeon	
1 - 4	14.6%
5 - 9	21.3%
10 - 14	22.5%
15 - 19	15.8%
20 - 24	12.1%
> 25	13.7%
Number of breast surgery per year	
< 25	24.7%
25 - 49	26.3%
50 - 74	16.5%
75 - 99	8.2%
> 100	24.3%

*: expressed as percentage of respondents.

**Figure 1:**
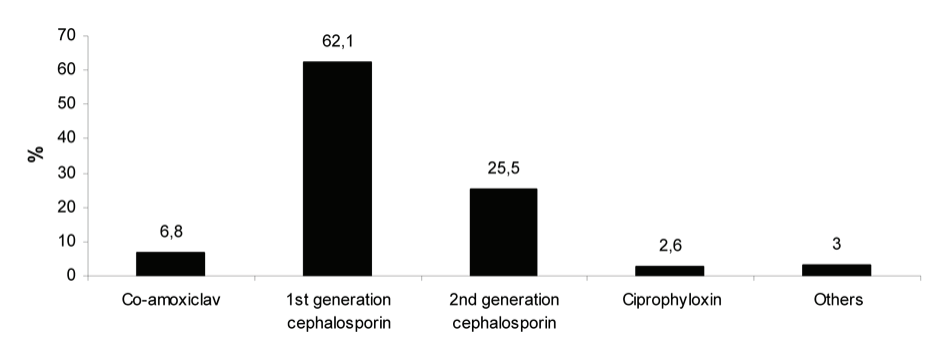
Distribution of the antibiotics selected for prophylaxis among surgeons.

As shown in Figure 1, the majority of respondents (62.1%) favor the use of first generation of cephalosporins for prophylaxis. Other surgeons select second generation of cephalosporins, co-amoxiclav, ciprophyloxin or other antibiotics (25.5%, 6.8%, 2.6%, and 3%, respectively). Most surgeons (69.4%) preferred single dose antibiotic for prophylaxis, while 15.3% of respondents prescribed second dose and others (15.3%) administrated prophylactic antibiotics beyond the 24-h postoperative period.

Despite the use of antibiotic prophylaxis SSI might develop. Most respondents (57.6%) encountered *Staphylococcus aureus* as cause of postoperative infection, while other surgeons reported that the pathogens most frequently implicated in SSIs were *Staphylococcus epidermidis* (28.8%) and other microorganism (13.6%) in spite of the use of prophylactic antibiotic.

A majority of Turkish surgeons (83.5%) preferred to use antibiotic prophylaxis if the patient has diabetes mellitus (Table 2). Most of respondents reported the use of antibiotic if the patient is elderly. As seen in Table 2, most respondent chose to antibiotic prophylaxis if patients received neo-adjuvant chemotherapy or radiation therapy. If the patients had prior breast reconstruction, 66.2% of the respondents reported the preference of antibiotic prophylaxis. In the presence of length of operation > 2 h, 76.8% of respondents preferred to use additional dose of the antibiotic. In addition, 91.5% of the respondents preferred to use antibiotic prophylaxis in the presence of immunodeficiency or immunosuppressive drug. Although most respondents (55%) reported the use of prophylactic antibiotic in the presence of surgical drain, others (45%) did not prefer to antibiotic prophylaxis. This difference was found to be statistically insignificant (P = 0.091).

**Table 2:** Surgeons’ Responses Regarding Antibiotic Prophylaxis in Accordance With Known Risk Factors of Surgical Site Infection

Risk factors	Percentage of respondents	P value
The presence of diabetes mellitus		
yes	83.5%	
no	16.5%	0.000
Older patient		
yes	58.8%	
no	41.2%	0.006
receiving immunosuppressive drug		
yes	91.5%	
no	8.5%	0.000
Neoadjuvant chemotherapy		
yes	75.9%	
no	24.1%	0.000
Neoadjuvant radiation therapy		
yes	70.6%	
no	29.4%	0.000
Prior breast reconstruction		
yes	66.2%	
no	33.8%	0.000
the use of drain		
yes	55.5%	
no	44.5%	0.091*
additional dose if operation duration > 2hrs		
yes	76.8%	
no	23.2%	0.000

*: not significant.

**Figure 2:**
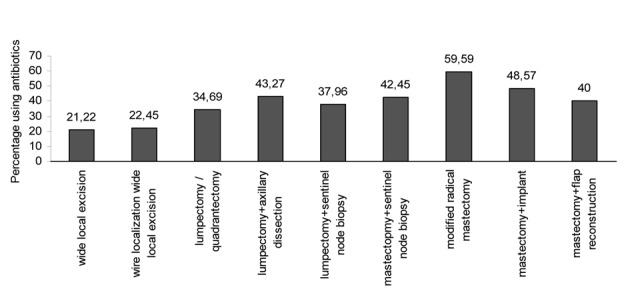
The percentages of surgeons using antibiotic prophylaxis for various breast surgical procedures.

The percentages of general surgeons who use antibiotic prophylaxis for the various surgical procedures are shown in Figure 2. A majority of the surgeons who perform mastectomy with or without breast reconstruction use prophylactic antibiotics. However, minority of the respondents prefer to use antibiotic prophylaxis in patients undergoing wide local excision.

## Discussion

The rates of SSI in breast surgery including axillary procedures vary from 1.4% to 38.3% depending on the type of surgical procedure: 1.5% for wide excision, up to 38% for mastectomy [1, 2, 4, 10-12, 16]. In British ALMANAC trial, the SSI rates were examined in patients with axillary dissection versus sentinel lymph node biopsy (SLNB) [17]. The SSI rates were 11% and 14% after SLNB and axillary dissection, respectively. Similarly, SSI rate was higher in breast cancer patients with axillary dissection (8%) than those with SLND (3%) in the American College of Surgeons Oncology Group Z0011 trial [18]. The rates seem to be a higher rate of infection than might be expected after other types of “clean surgery”. These high rates of post-operative infections provide the consideration of antibiotic prophylaxis even though breast surgery is considered “clean” procedure.

On the other hand, there is no clear evidence from published data for the benefit of antibiotic prophylaxis in breast cancer surgery. The pathogens most frequently implicated in postoperative breast infections are Staphylococci which are often sensitive to cephalosporins and co-amoxiclav. Several surgeons have been used the prophylaxis with preoperatively on dose of an intravenously administrated antibiotic with anti-staphylococci activity. However, in a study from Mexico bacteria isolated were mainly gram-negative, which is not expected with breast surgery [2].

Although there is no consensus on the use of prophylactic antibiotics for breast cancer surgery, peri-operative antibiotics have been used to decrease the infection rates. In some studies, the results showed that preoperative antibiotic prophylaxis significantly reduces the incidence of SSI in patients undergoing breast cancer surgery [1, 2, 4-7]. In a nested case-control study by Vilar-Compte and co-workers, the rate of SSI after breast surgery was 25.8% [2]. In their study, the multivariate analysis demonstrated that obesity, pre-operative chemotherapy or radiotherapy, radical surgery, length of drain stay > 20 days and need of a second drain insertion were related to the development of SSI.

Some surgeons limit the use of antibiotic to high-risk patients. The factors associated with postoperative infection in breast surgery are as follows: obesity, neoadjuvant chemotherapy or radiation therapy, prolonged closed suction drainage, second drain placed, diabetes mellitus, immunodeficiency, steroid use, hematoma, seroma, length of surgery, type of surgery, immediate breast reconstruction, advanced age, and smoking [2-6, 13, 19-22]. The authors have suggested that antibiotic prophylaxis is beneficial for patients with high risk for SSI after breast cancer surgery. Routine antibiotic prophylaxis is not necessary for patients not at risk of SSI, because the rate of SSI in these patients is low [3, 5, 13, 22]. Interestingly, recent meta-analysis showed that antibiotic prophylaxis is not independent protective factor. The administration of antibiotic should be taken into consideration if other risk factors are accompanied [13].

It is clear that the published data have demonstrated a lack of consensus regarding antibiotic prophylaxis as well as risk factors associated with SSI after breast cancer surgery. Moreover, recent two nationwide surveys from United Kingdom and United States of America have shown that there is no consensus about the use of antibiotic prophylaxis in breast surgery among surgeons [14, 15]. We also performed the nationwide survey concerning the use of antibiotic prophylaxis. As shown in the findings of our survey, there is a lack of consensus in optimal use of antibiotic prophylaxis among Turkish surgeons. The antibiotic most commonly selected in our survey was cephalosporins (87.6%) followed by co-amoxiclav (6.8%). Our findings differ from English surgeons who prefer to use of co-amoxiclav [14]. In the survey from US, 99% of surgeons utilized cephalosporins as preoperative antibiotic prophylaxis in breast surgery requiring drains [15].

The survey among English surgeons considered only the use of antibiotic prophylaxis for various breast surgical procedures [14]. The majority of English breast surgeons use antibiotic prophylaxis in breast reconstruction, while about 30% of the surgeons who perform breast surgery without reconstruction use prophylactic antibiotic. In our survey we evaluated the relationship between the choice of antibiotic prophylaxis and the known risk factors for SSI like diabetes mellitus, older patient, neoadjuvant chemotherapy, use of immunosuppressive drug, use of surgical drain as well as type of breast cancer surgery. The results of our survey demonstrated that the use of prophylactic antibiotic was seen to be strongly dependent on patient’s age, type of operation, length of operation > 2 h, receiving preoperative chemotherapy or radiotherapy, the presence of co-morbidity of the patient including diabetes mellitus.

Some surgeons prefer postoperative prophylaxis for patients with drains after breast and/or axillary surgery to prevent SSI. Pre- and post-operative prophylactic antibiotics are used in patients undergoing mastectomy, surgical drain placed, immediate reconstruction or receiving prior chemotherapy or radiation therapy [1-5, 13, 19, 20, 22]. It is well known that closed-suction drainage after mastectomy and/or axillary dissection is accepted to prevent seroma formation. However, there is no consensus regarding the role of perioperative antibiotic prophylaxis in breast surgery utilizing drainage tubes. According to the studies by Felippe et al and Lanier et al, the use of drains after breast and axillary surgery is one of the significant risk factors for the development of SSI [23, 24]. Moreover, increased risk of SSI can be associated with longer drain duration [13, 19]. Looking at the results of our survey, the drain placement did not significantly influence prophylactic antibiotic usage. On the contrary, the survey of American Society of Breast Surgeons showed that 86% of the surgeon “always” administrated antibiotic prophylaxis in the breast surgery requiring drains [15].

Both the retrospective and prospective published studies and the surveys have demonstrated that there is a lack of consensus regarding the optimal use of antibiotic prophylaxis in breast cancer surgery requiring drains. Therefore further studies are needed to focus on antibiotic prophylaxis in breast surgery requiring drain placement. Besides, SSI should be defined according to the new criteria of the Centers for Disease Control and Prevention (CDC) [25]. More recently, Degnim and coworkers have showed that the rates of SSI after breast and axillary surgery are reduced threefold when 2010 CDC reporting guidelines are used [26].
